# Fangcang Shelter Hospital in Wuhan: A radiographic report on a cohort of 98 COVID-19 patients

**DOI:** 10.7150/ijms.48074

**Published:** 2020-07-30

**Authors:** Tingting Guo, Xiaoming Liu, Cihao Xu, Jiazheng Wang, Lian Yang, Heshui Shi, Meng Dai

**Affiliations:** 1 Department of Radiology, Union Hospital, Tongji Medical College, Huazhong University of Science and Technology, Wuhan 430022, China.; 2 Hubei Province Key Laboratory of Molecular Imaging, Wuhan 430022, China.; 3 MSC Clinical & Technical Solutions, Philips Healthcare, Beijing, 100000, China.

**Keywords:** Coronavirus disease 2019 (COVID-19), CT, ground-glass opacity (GGO)

## Abstract

**Objectives:** To present the temporal changes of CT manifestations in COVID-19 patients from a single fangcang shelter hospital and to facilitate the understanding of the disease course.

**Materials and Methods:** This retrospective study included 98 patients (males: females, 43:55, mean year, 49±12 years) with confirmed COVID-19 at Jianghan fangcang shelter hospital admitted between Feb 05, 2020, and Feb 09, 2020, who had initial chest CTs at our hospital. Radiographic features and CT scores were analyzed.

**Results:** A total of 267 CT scans of 98 patients were evaluated. Our study showed a high median total CT score of 7 within the first week from symptom onset, peaked in the 2^nd^ week at 10, followed by persistently high levels of CT score with 9.5, 7 and 7 for the week 3, 4, and >4, respectively, and a prolonged median disease course (30 days, the median interval between the onset of initial symptoms and discharge). Ground-glass opacity (GGO) (58%, 41/71) was the earliest and most frequent finding in week 1. Consolidation (26%, 14/53) and mixed pattern (40%, 21/53) were predominant patterns in 2^nd^ week. GGO and reticular were the main patterns of later phase CT scans in patients with relatively advanced diseases who had longer illness duration (≥4 weeks). Among the 94 CT abnormalities obtained within 3 days from the twice RT-PCR test turned negative, the mixed pattern was mainly presented in patients with disease duration of 2-3 weeks, for GGO and reticular were common during the whole course.

**Conclusion:** Discharged patients from fangcang shelter hospital demonstrated a high extent of lung abnormalities on CT within the first week from symptom onset, peaked at 2^nd^ week, followed by persistence of high levels and a prolonged median disease course. GGO was the predominant pattern in week 1, consolidation and mixed pattern in 2^nd^ week, whereas GGO and reticular patterns in later stages (≥4 weeks).

## Introduction

Since the outbreak of coronavirus disease 2019 (COVID-19) in Wuhan in December 2019, it has spread worldwide and infected more than 3 855 788 people by 9 May 2020 1. With the high rate of human transmission and a mortality rate of 2.9% in Hubei province compared to that of 0.4% outside of the region 2, the disease has been recognized as a pandemic by the WHO on Mar 11, 2020 3.

In Wuhan, 16 fangcang shelter hospitals have been built-up to hospitalize, treat and monitor the patients with confirmed COVID-19, such that the limited medical resources could distribute for mild-type, common-type, severe-type, and fatal severe-type patients. By March 10, more than 12,000 patients 4 had been treated uniformly according to the standard protocols 5, and all the fangcang shelter hospitals in Wuhan had been closed due to the recent relief of the epidemics.

Previous studies have revealed the typical radiological imaging features of COVID-19 6-12. However, it remains understudied how the chest CT manifestations evolve in the patients of fangcang shelter hospitals, who were hospitalized according to uniform criteria 5 and who went through more regular examinations and uniform treatments 5. In the meanwhile, as will be introduced below, the medical intervention in the fangcang shelter hospitals could be potentially practiced in a self-isolation environment such as at home. Therefore, the purpose of this study was to determine the temporal patterns of CT manifestations in COVID-19 patients from a single fangcang shelter hospital and to facilitate the understanding of the disease course.

## Methods

The Jianghan fangcang shelter hospital in this study was operated by our hospital. This retrospective study was approved by the Ethics of Committees of our hospital. Only the anonymous data was collected, and informed consent for this retrospective study was waived.

### Admission to, treatment at, and discharge from the Fangcang Shelter Hospitals

The admission criteria for fangcang shelter hospitals were^13^: 1) patients with confirmed COVID-19; 2) Clinical classifications were mild-type (clinical symptoms without CT abnormity) or common-type (clinical symptoms with CT abnormity); 3) no respiratory, cardio, or mental diseases, no other basic diseases; 4) 16-65 years old with self-care ability; 5) negative test results of influenza virus assays.

The treatment protocol and discharge criteria followed 'The 6th Edition of Pneumonia diagnosis and treatment plan for new coronavirus infection' formulated by National Health Commission of the People's Republic of China 5.

For treatment: 1) Oseltamivir: 75 mg/tablet, 2 tablets each time, 2 times a day, less than 10 days; 2) Abidor 14: 200 mg for adults, 3 times a day, less than 10 days; 3) Moxifloxacin: 0.4 g for adults, once a day, less than 7 days; 4) LianHua QingWen Formular: 1.4 g each time, three times a day, less than 10 days.

For discharge: 1) afebrile for more than 3 days; 2) respiratory symptoms markedly improved; 3) improvement in the radiological abnormalities on chest CT (Reduction in density and range of lesions); 4) two successive negative PCR tests with at least 24 h interval.

### Patients

We retrospectively reviewed the clinical records of patients for Jianghan fangcang shelter hospital who were admitted between February 05, 2020, and February 09, 2020, and the initial symptoms and symptom onset time were recorded. The inclusion criteria were: 1) patients had initial chest CT at our hospital (hence the integrity of the clinical and imaging records) were included in the study; 2) patients had been discharged from Jianghan fangcang shelter hospital by the censored date (March 09, 2020).

### CT image acquisition

To minimize motion artifacts, patients were instructed on breath-holding in the supine position. All chest CT scans were performed within a single inspiratory phase on two commercial multi-detector CT scanners (uCT550, Shanghai, China, Shanghai United Imaging Healthcare; Aquilion16-slice, Toshiba Medical Systems, Tochigi, Japan). For CT acquisition, the tube voltage was fixed at 120 kVp, the pitch of 1.075, scanning interval of 0.55 mm, rotation time of 0.6s, and collimator width of 22 mm. Low-dose scanning mode with an automatic tube current of 40 mAs and dose level at 1 was performed to reduce the radiation risk of the patients. CT images were reconstructed with a matrix size of 512 × 512 (thickness of 1.5 mm and increment of 1.5 mm) in transverse slice orientation. The images were acquired for lung (window width, 1,000-1,500 HU; window level, -700 HU) and mediastinal (window width, 350 HU; window level, 35-40 HU) settings.

### CT Image Interpretation

Image analysis was reviewed by three radiologists (H. S, L. Y, and M. D who had 30, 22 and 10 years of experience in thoracic radiology, respectively), using the institutional digital database system (Vue PACS, version 11.3.5.8902, Carestream Health, Canada). Decisions were reached by consensus when there was a disagreement between the three radiologists.

Depending on the time between symptoms onset and the CT scan, all the CT scans were divided into 5 groups: Group 1 (scans obtained the first week from symptom onset), Group 2 (2^nd^ week), Group 3 (3^rd^ week), Group 4 (4^th^ week), and Group 5 (>week 4).

Predominantly CT pattern, including ground-glass opacity (GGO), consolidation pattern, reticular pattern, and the mixed pattern (the combination of GGO, consolidation, and reticulation) 6, 8, 9, 15. Distribution of abnormal opacities was recorded mainly as three types: subpleural (involving mainly the peripheral one-third of the lung), random (without predilection for subpleural or central regions), and diffuse (continuous involvement without respect to lung segments) 6. Additionally, the presence of pleura effusion, mediastinal lymphadenopathy (axil diameter >1.0 cm) was also noted.

A total score of 0-25 was obtained as the summation of 5 lobes (score 0-5 for each lobe: score 0, no involvement; score 1, <5% involvement; score 2, 6%-25% involvement; score 3, 26%-49% involvement; score 4, 50%-75% involvement; score 5, >75% involvement) 16.

### Statistical analysis

The median values of total CT scores were plotted against time since symptom onset. The temporal changes in CT abnormity patterns and the distribution of lung abnormalities were analyzed. Manne-Whitney U test was used for the difference between the median values of CT lung quantification in different groups, and the Chi-square test was applied to compare the frequency of CT patterns in different groups. A P-value of < 0.05 was considered statistically significant. Statistical analysis was performed by using statistical software (SPSS version 18, IBM, New York, USA).

## Results

### Patients

Between February 05, 2020, and February 09, 2020, a total of 125 patients for Jianghan fangcang shelter hospital who performed the initial CT scans in our hospital were conducted. Another 27 patients were excluded because they were transferred to designated hospitals due to failure to meet discharge criteria. The remaining 98 patients (43 males and 55 females) with confirmed COVID-19 were included in this study, all of whom had been discharged by the censored date. The mean age of the patients was 49 ± 12 years old (range 15-67 years). The most common symptoms at the onset of illness were fever (82%) and cough (52%). Most fever patients had either low-grade (38%, 37.4-38 °C) or moderate fever (32%, 38.1-39.0 °C). All patients were discharged with a median hospitalized period of 18 (interquartile range: 14-22) days, range 9-33 days. Median time from symptom onset to discharge was 30 (interquartile range: 26-36) days, range 15-49 days. The demographic characteristics, initial symptoms, and clinical records were summarized in **Table 1**.

### General CT manifestations

**Figure 1:**
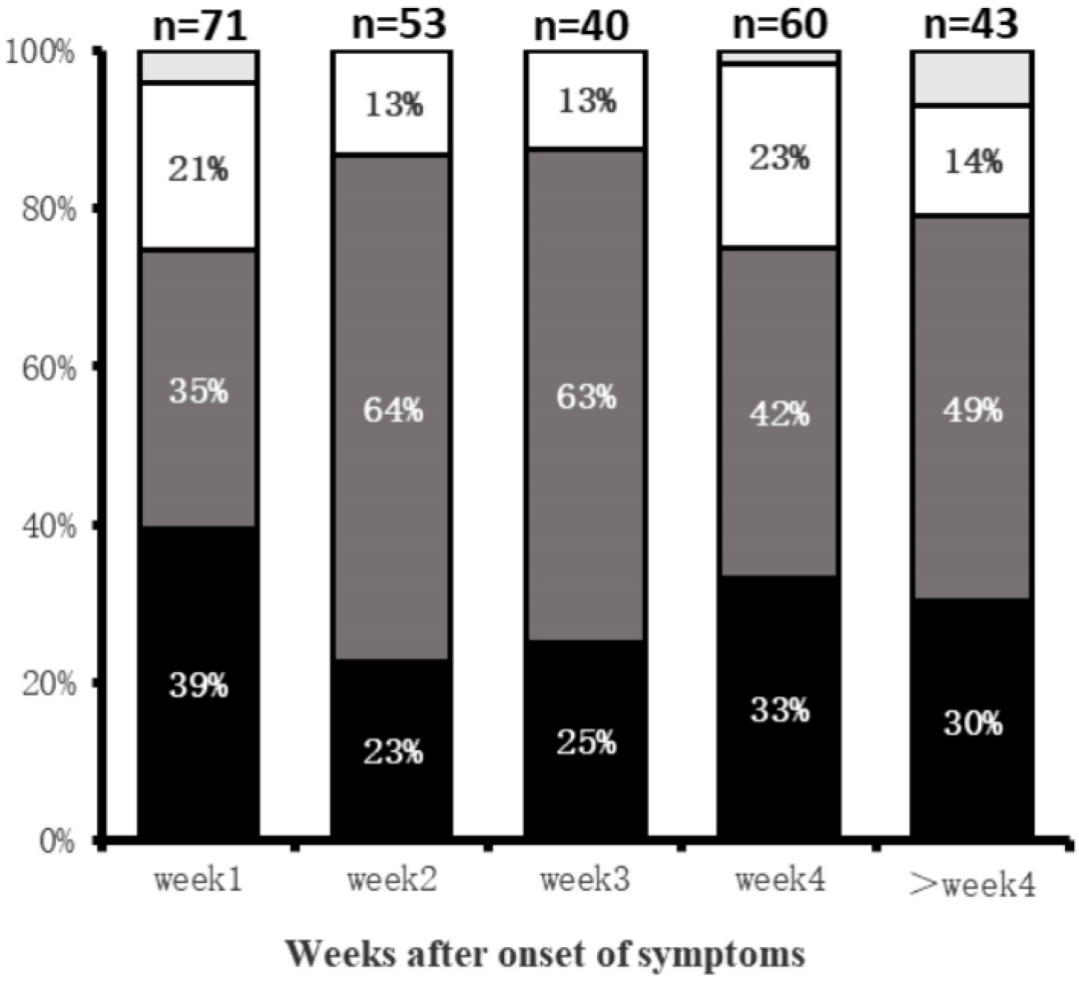
Distributions of Lung abnormities at various times in weeks after symptoms onset. Categories with a percentage of ≥10% are shown. Black = subpleural; dark gray = diffuse; white = random; light gray = no lesion.

Among the 98 patients, 40 had twice CT scans, 45 had three CT scans, and the other 13 had four CT scans. A total of 267 CT scans was acquired and evaluated. Group 1 (n=71), Group 2 (n=53), Group 3 (n=40), Group 4 (n=60), and Group 5(n=43). For Group 1, the predominant demonstrations were subpleural (39%, 28/71 patients) and diffuse (35%, 25/71) lung abnormalities. Lung abnormalities tended to be most diffuse (64%, 34/53) in Group 2 **(Fig. 1)**. Bilateral lung involvement was commonly observed during the whole course of the disease (lung involvement 76%-90%) with the highest percentage of 90% (36/40 patients) in Group 3 **(Table 2)**.

### Temporal changes in CT scores

**Figure 2:**
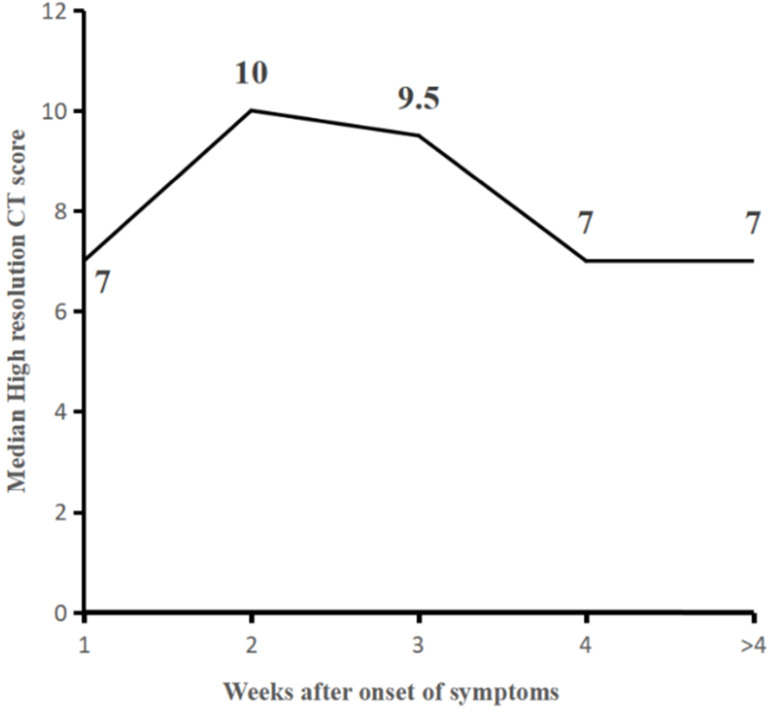
Line graph showing the median CT scores at various time points in weeks after onset of symptoms. CT scores peaked at 2nd week of illness, with a platform period after that and slow decline substantial scores after the 3rd week. The median score between Group1 and 2, Group 3, and 4 were statistically significant, respectively (P=0.015, P=0.006).

The median values of total CT score were 7 (interquartile range: 3-10) for the first week (Group 1), higher than reported in the literatures (6, 10, 16), which slowly increased to a peak value of 10 (interquartile range: 4-14, p< 0.05, compared to that of within the first week) at the second week (Group 2) and started to decrease substantially afterward. The median values of total CT scores were 9.5 (7, 14), 7 (4, 10), and 7 (4, 11) for the Groups 3, 4, and 5, respectively. Radiological improvement occurs after the third week, with a statistically significant decrease in median scores between Group 3 and Group 4 (p=0.006) **(Table 2, Fig. 2).**

### Temporal changes of predominant imaging patterns

**Figure 3:**
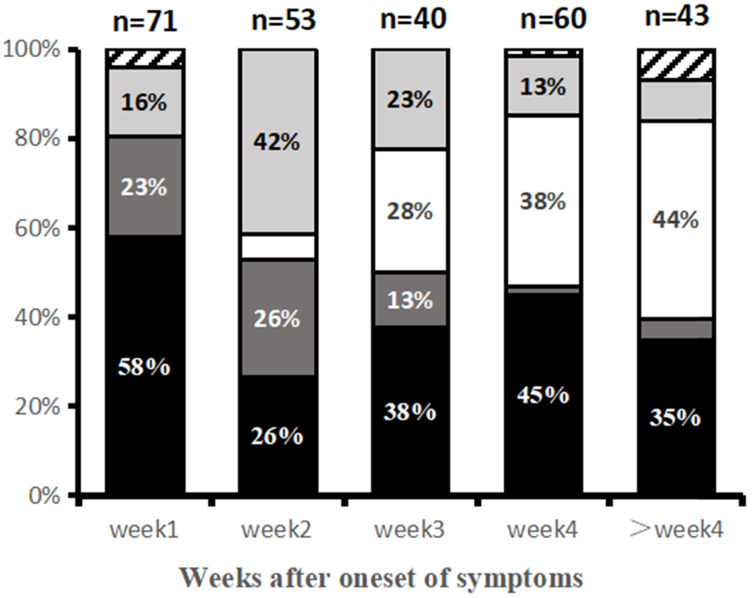
Main CT patterns at various time points since symptom onset. Categories with Percentage ≥10% are shown. Black = ground-glass opacities (GGO) pattern; Dark gray = consolidation pattern; light gray = mixed pattern; white = reticular pattern; striped = normal.

Predominant CT image patterns changed over time as the disease progressed. Within the first week (Group 1) after symptom onset, the GGO pattern was the earliest and most common imaging finding (58%, 41/71 patients). In the second week (Group 2), consolidation and mixed pattern developed to the predominant patterns with demonstration frequencies of 26% (14/53) and 40% (21/53). In the third week of illness (Group 3), imaging patterns became more diverse, including GGO (38%, 15/40 patients), reticular pattern (28%, 11/40), and mixed pattern (23%, 9/40). GGO and reticular patterns were the main imaging patterns in Group 4 (4^th^ week) and Group 5 (>4 weeks) **(Table 2, Fig. 3).**

**Figure 4:**
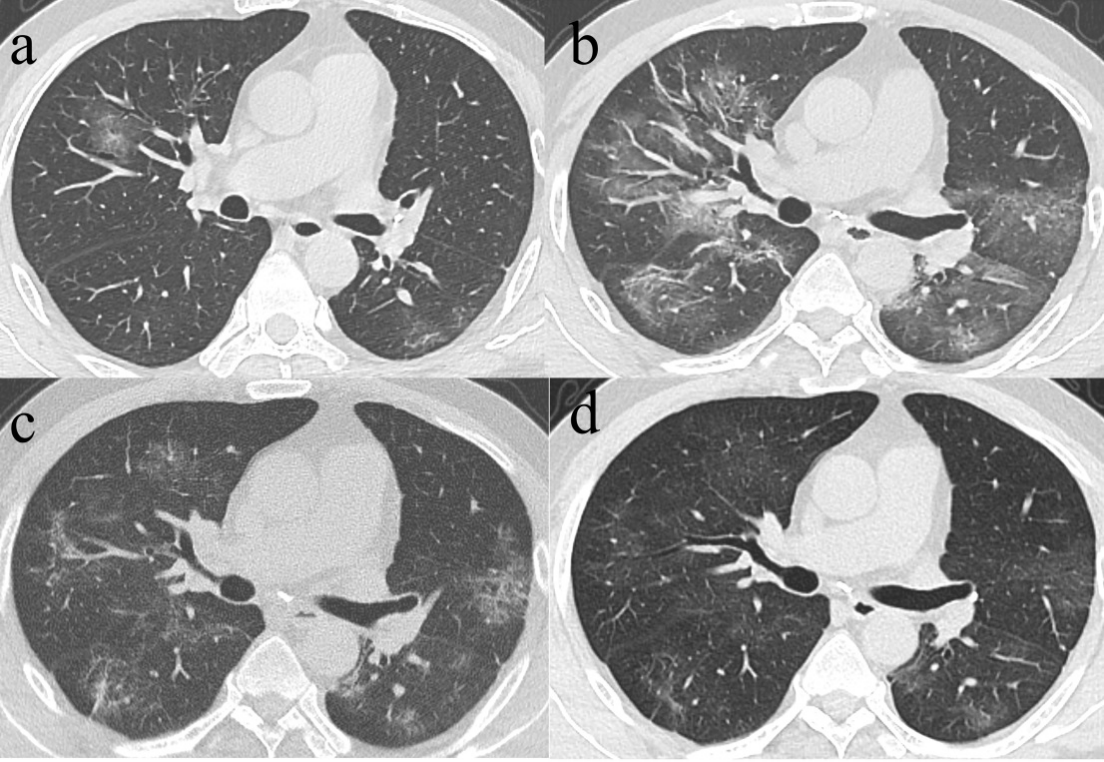
** Series CT scans in a 61-year-old man with COVID-19 pneumonia. a.** Scan obtained on illness days 2 showed GGO that affected the right middle lobe and left lower lobe. **b**, Scan obtained on illness days 9 showed an increased extent of GGO with little parenchymal bands.** c.** Scan obtained on illness days 19 showed absorption of abnormalities, with GGO and little parenchymal bands. **d.** Scan obtained on illness days 33 showed obvious absorption of abnormalities. Only GGO could be observed. The patient was discharged on illness days 36.

**Figure 5:**
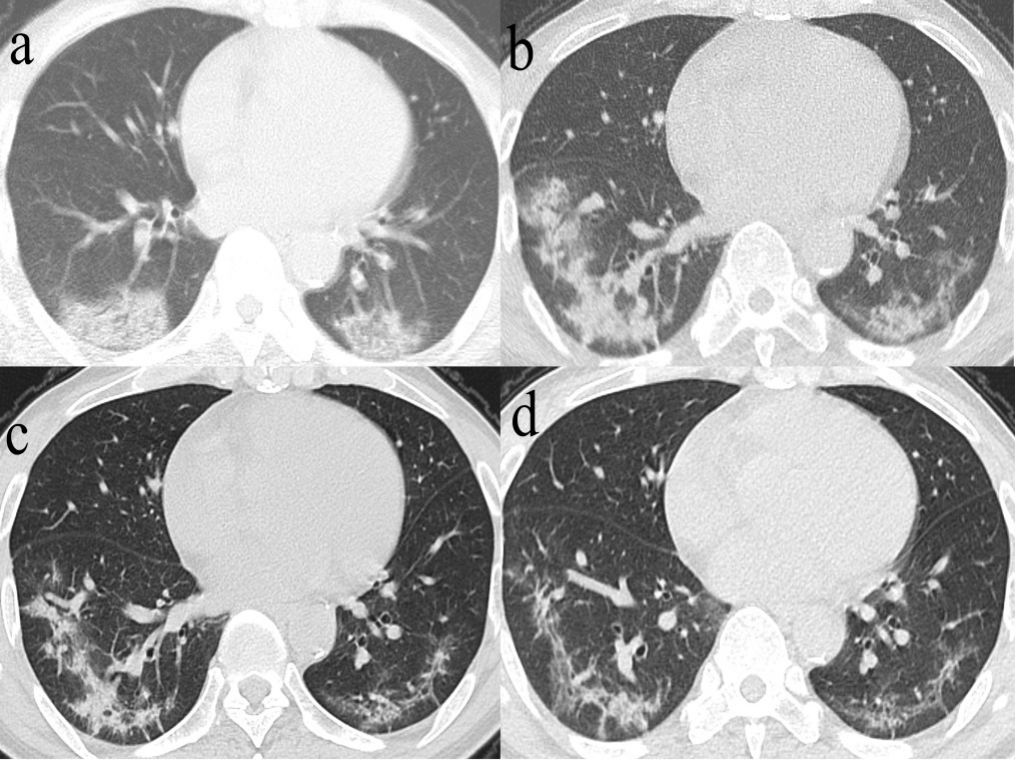
** Series CT scans in a 65-year-old man with COVID-19 pneumonia. a.** Scan obtained on illness days 4 showed consolidation affected in both lower lobes. **b.** Scan obtained on illness days 18 showed consolidation with an increased extent. **c.** Scan obtained on illness days 27 showed a predominantly mixed pattern with bandlike consolidation and parenchymal bands in both lower lobes. **d.** Scan obtained on illness days 36 showed a predominantly reticular pattern with interlacing line shadows mainly in both lower lobes. The patient was discharged on illness days 39.

Among the 71 CT scans in Group 1 who had their initial CT taken within the 1^st^ week of symptom onset, 8 patients with repeated examinations or no lesions were excluded, hence, 63 patients demonstrated CT abnormalities, including GGO in 38 patients, consolidation in 15 patients, and mix pattern for 10 patients. For the 38 patients with initial GGO, 37 presented varying residual lesions in the final scans, including GGO pattern [17 patients, median CT score: 3 (1, 7)]** (Fig. 4)**, reticular pattern [18 patients, 9 (5, 13)] and mixed pattern [2 patients, 8 (5, 11)]. Noticeably, the median CT score of patients with the final reticular pattern was higher than that of patients with final GGO (p=0.004). Among the 38 patients with initial predominant GGO, 17 had final CT scan at discharge also demonstrated predominant GGO, while the median CT scores had significant differences between the initial [median CT score: 5 (3, 9)] and final CTs [median CT score: 3 (1, 7)] (p=0.033). On the other hand, 18 of the 38 patients had their initial predominant GGO turned to the reticular pattern in the final scans, for whom no significant differences were observed between the initial [median CT score: 8.5 (3.75,10.25)] and the final CT scores [median CT score: 9 (5,13)] (p=0.253). For the 15 patients with initial consolidation, residual lesions in the final scans were GGO in 9 patients [median CT score: 4 (2, 7)] and reticular in 4 patients [median CT score: 7 (3, 9.5)]** (Fig. 5).** For the 10 patients with initially mixed patterns, residual lesions in the final scans were GGO in 2 patients and reticulation in 7 patients [median CT score: 8 (2, 11)] **(Table 3).**

94 out of 98 patients had their last CT scans obtained within 3 days from the twice PCR test that turned negative. The last CT scans were obtained more than 3 days from the PCR turning negative for the other 4 patients. Among the last CT scans for these 94 patients, the mixed pattern was mainly presented in patients with a disease duration of 2-3 weeks, for whom GGO and reticular were consistently the most common CT abnormalities during the whole course. Complete lesion absorption was observed for 4 (4/94) patients only. Substantial residual lesions (median CT score ≥5) mainly existed in patients with the disease course of greater than 3 weeks **(Table 4)**.

Four of the 98 patients (4%) developed mild unilateral (3/4 patients) and bilateral (1/4) pleural effusion at 6, 10, 23, and 27 days since symptom onset. Mediastinal lymphadenopathy was not observed in our study.

## Discussion

In this study, serial CT scans obtained in the cohort of Jianghan fangcang shelter hospital were analyzed to explore the temporal imaging changes from symptom onset. Lung injury in CT demonstrations developed remarkably after symptom onset and peaked in the second week, followed by a slow decrease of total CT scores. Remarkably, the median CT score in the 1^st^ week from symptom onset was higher than those reported previously for COVID-19 patients 10, 16 and even SARS 6 patients. The peak of the CT score appeared in the 2^nd^ week since symptom onset in our cohort, which was consistent with 6-11 days reported by Pan et al. 16 and Wang et al. 10 with COVID-19 patients and the 2^nd^ week reported by Ooi et al. 6 for SARS patients, and which was, however, controversial to the rapid lung injury as presented for SARS and MERS 6, 17. The high median CT score in the 1^st^ week from symptom onset in our cohort was accompanied by longer median disease duration of 30 (26,36) days and hospitalized 18 (14,22) days, in contrast to the median disease duration of 25 (10, 56) days and hospitalized period of 18 (5, 43) days reported by Wang et al. 10 and the mean disease duration of 19 ± 4 days and hospitalized period of 17±4 days as reported in Pan et al. 16. These observations of high initial CT score and longer disease duration might be attributed to the older cohort in our study (49 ± 12 years) as compared to the cohort ages of 40 ± 9 years in Pan et al. 16 and 45 ± 14 years in wang et al. 6, 10. The patients reported in SARS 6 and MERS 17 studies were 42.5 ± 12.2 years old and 44.2 ± 16.1 years old, respectively, which might have led to a shorter disease course and, hence, the earlier CT score peak when compared to the cases in our cohort. The differences in treatments may be another cause of varying disease course. Oral antiviral agents, complaint management and symptomatic treatment were the main treatments in fangcang shelter hospitals 5, while oxygen treatment/high-flow nasal cannula, mechanical ventilation, glucocorticoids therapy and emergency extracorporeal membrane oxygenation (ECMO) were commonly used for severe patients in designated hospitals 2, 18. Therefore, the similar peak time, despite the higher initial CT score, when compared to the previous reports might have revealed the progressive nature of the disease at the absence of complicated treatments in the fangcang shelter hospitals, which was also evidenced by the slow decrease and persistent high CT scores after the 2^nd^ week in our study that was suggestive of the slow absorption of the COVID-19 lung lesions. To confirm this speculation, we will review the data from multiple fangcang shelter hospitals for larger sample size and extended monitoring.

In this work, predominant GGO was the commonest and earliest imaging finding within the first week after symptom onset, while consolidation became predominant in the second week. Predominant GGO and consolidation have also been widely reported in previous studies as the common lung abnormalities within the first 2 weeks after symptom onset in COVID-19, SARS or MERS 6, 9-11, 16, 17, 19. A recent postmortem pathology study 20 for a patient with a 15-day disease course revealed the exudative change in the lung and the absence of severe fibrosis, suggesting diffuse alveolar damage in response to acute lung injury, which was supportive to the observed CT manifestations in the first 2 weeks. The severity and distribution of gray-white patch lesions in the lung in the pathology had explained the predominant GGO in the early stage. The eventual consolidation was considered a result of cellular fibro-myxoid exudates in alveoli 21.

Radiological improvement occurred after the third week. For later stages (≥4 weeks), predominant GGO and reticular pattern became common findings, inconsistency with the CT demonstrations of SARS 6, 22, 23 or ARDS 24. However, Wang et al. 10 indicated GGO and mixed pattern were the common findings in the later stage of COVID-19 pneumonia, the cause of this difference remaining unclear. Particularly, the reticular pattern experienced a persistent increase since the 2^nd^ week in our study, and the lung involvement of GGO had an obvious decrease in the second week and increased after that. Besides, GGO and reticular were the main patterns of later phase CT scans in patients with relatively advanced diseases who had longer illness duration (≥4 weeks). Elevation of GGO in later stages had also been reported before for both COVID-19 10 and SARS 6. Noticeably, patients with predominant reticular pattern in the late stages had significantly higher late-stage CT scores than those with late-stage predominant GGO, implying the appearance of GGO in later stage suggestive of better recovery of the illness 6, 10. In contrast, substantial residual reticular pattern in later stage might indicate the possible development of fibrosis 25 or secondary organizing pneumonia 26. Irreversible fibrosis might be too early to label due to uncharted nature history 6. Our observation raised the necessity of long-term follow-up in chest CT and concomitant functional tests to determine the long-term pulmonary sequelae of COVID-19 22.

Although all the patients in this cohort matched with the discharge criteria by the end of the study, varying residual lesions were still observed via CT. In light of a recent report of 4 discharged patients with recurrent positive RT-PCR results 27, follow up of these patients is also necessary from the perspective of epidemic prevention.

The current study was mainly limited as no severe patients were included.

In conclusion, our study showed a high median total CT score of 7 within the first week from symptom onset, peaked at 2nd week, followed by persistence of high levels and a prolonged median disease course. GGO was the predominant pattern in week 1, consolidation and mixed pattern in 2^nd^ week, whereas GGO and reticular patterns in later stages (≥4 weeks).

## Highlights

- A high median total CT score of 7 within the first week from symptom onset, peaked at 2^nd^ week, followed by persistence of high levels and a prolonged median disease course;
- Ground-glass opacity (GGO) was the earliest and most frequent finding in week 1. Consolidation and mixed pattern were predominant patterns in 2^nd^ week. GGO and reticular were the main patterns in the later stages of the disease;
- Among the 94 patients with CT abnormities obtained within 3 days from the twice RT-PCR test turned negative, GGO and reticular were common during the whole course.

## Figures and Tables

**Table 1:** Characteristics of the patient cohort

All patients (n=98)	
**Age (y)**	49 ± 12 (15-67)
**Gender**	
Male	43 (44%)
Female	55 (56%)
**Initial symptoms**	
Cough	51 (52%)
Fever	80(82%)
Low grade fever (37.4 - 38.0 °C)	37 (38%)
Moderate grade fever (38.1 - 39.0 °C)	31(32%)
High grade fever (>39.0 °C)	12 (12%)
Fatigue	35 (36%)
Myalgia	31 (32%)
Chest tightness	47 (48%)
Anorexia	50 (51%)
Diarrhoea	26 (26%)
Headache	7 (7%)
The hospitalized period (d)	18 (14,22)
The interval between the onset of initial symptoms and discharge (d)	30 (26,36)

**Note:** Quantitative data were presented as mean ± standard deviation (minimum-maximum), while the counting data were presented as count (percentage of the total). The hospitalized period and the interval between the onset of initial symptoms and discharge were presented as median (25%, 75%).

**Table 2:** Temporal changes of lung abnormities in CT scans since symptom onset

	Group 1 (n=71)	Group 2 (n=53)	Group 3 (n=40)	Group 4 (n=60)	Group 5 (n=43)
**Distribution of pulmonary lesions**					
No lesion	3 (4%)	0 (0%)	0 (0%)	1 (2%)	3 (7%)
subpleural	28 (39%)	12 (23%)	10 (25%)	20 (33%)	13 (30%)
Random	15 (21%)	7 (13%)	5 (13%)	14 (23%)	6 (14%)
Diffuse	25 (35%)	34 (64%)	25 (63%)	25 (42%)	21 (49%)
**Involvement of the lesions**					
No lesion	3 (4%)	0 (0%)	0 (0%)	1 (2%)	3 (7%)
unilateral lung	14 (20%)	10 (19%)	4 (10%)	9 (15%)	2 (5%)
bilateral lung	54 (76%)	43 (81%)	36 (90%)	50 (83%)	38 (88%)
**Patterns**					
GGO	41 (58%)	14 (26%)	15 (38%)	27 (45%)	15 (35%)
Consolidation	16 (23%)	14 (26%)	5 (13%)	1 (2%)	2 (5%)
Reticular	0 (0%)	3 (6%)	11 (28%)	23 (38%)	19 (44%)
Mixed	11 (15%)	21 (40%)	9 (23%)	8 (13%)	4 (9%)
Number of involved lobes	5 (2,5)	5 (2,5)	5 (4,5)	5 (2.25,5)	4 (3,5)
Total Scores	7 (3,10)	10 (4,14)#	9.5 (7,14)	7 (4,10)#	7 (4,11)

**Note:** The counting data were presented as count (percentage of the total). Total scores were presented as median (25%, 75%). Manne-Whitney U test showed the median total CT scores had significant difference between group 1 (0-7 days) and group 2(8-14 days, P=0.015), group 3 (15-21 days) and group 4 (22-28 days, P=0.006). There was no statistical significant difference between group 2 and group 3(P=0.529), group 4 and group 5 (>28 days, P=0.917).

**Table 3:** Changes between the initial and final CT demonstrations

Initial CT patterns	GGO (n=38)				Consolidation (n=15)				Mixed (n=10)		
Final CT patterns	No lesion	GGO	Reticular	Mixed	No lesion	GGO	Reticular	Mixed	No lesion	GGO	Reticular
Number (%)	1 (2.6%)	17 (44.7%)	18 (47.3%)	2 (5.2%)	1 (6.6%)	9 (60%)	4 (26.6%)	1 (6.6%)	1 (10%)	2 (20%)	7 (70%)
Total scores	0 (-)	3 (1,7)	9 (5,13)	8 (5,11)	0 (-)	4 (2,7)	7 (3,9.5)	2 (-)	0 (-)	6 (6,6)	8 (2,11)

**Note:** The counting data were presented as count (percentage of the total). Total scores were presented as median (25%, 75%).

**Table 4:** CT abnormities obtained within 3 days from the twice RT-PCR test that turned negative

	week 1 (n=0)	week 2 (n=9)	week 3 (n=16)	week 4 (n=42)	> week 4 (n=27)
No lesion	0 (0%)	0 (0%)	0 (0%)	1 (2%)	3 (11%)
**Patterns**					
GGO	0 (0%)	3 (33%)	6 (38%)	16 (38%)	12 (44%)
Consolidation	0 (0%)	0 (0%)	0 (0%)	0 (0%)	1 (4%)
Reticular	0 (0%)	3 (33%)	6 (38%)	18 (43%)	10 (37%)
Mixed	0 (0%)	3 (33%)	4 (25%)	7 (17%)	1 (4%)
Total scores	0 (0%)	3 (33%)	8 (3,11)	8 (4,10)	5 (3,9)

**Note:** The counting data were presented as count (percentage of the total). Total scores were presented as median (25%, 75%).

## Abbreviations

COVID-19: Coronavirus disease 2019
SARS-CoV-2: Severe acute respiratory syndrome coronavirus 2
SARS: Severe acute respiratory syndrome
MERS: the Middle East respiratory syndrome
GGO: Ground-glass opacity
